# Carbohydrate Restriction with or without Exercise Training Improves Blood Pressure and Insulin Sensitivity in Overweight Women

**DOI:** 10.3390/healthcare9060637

**Published:** 2021-05-27

**Authors:** Shengyan Sun, Zhaowei Kong, Qingde Shi, Haifeng Zhang, On-Kei Lei, Jinlei Nie

**Affiliations:** 1 Institute of Physical Education, Huzhou University, Huzhou 313000, China; antun0605@163.com; 2 Faculty of Education, University of Macau, Macao 999078, China; mb54871@connect.um.edu.mo; 3 School of Physical Education and Sports, Macao Polytechnic Institute, Macao 999078, China; qdshi@ipm.edu.mo (Q.S.); jnie@ipm.edu.mo (J.N.); 4 College of Physical Education, Hebei Normal University, Shijiazhuang 050024, China; hbnuzhanghaifeng@sina.com

**Keywords:** low-carbohydrate diet, exercise, cardiometabolic health, appetite regulating hormones, obesity

## Abstract

Objective: The purpose of this study was to evaluate the effects of a 4-week low-carbohydrate diet (LC) with or without exercise training on cardiometabolic health-related profiles in overweight/obese women. Methods: Fifty overweight/obese Chinese women (age: 22.2 ± 3.3 years, body mass index (BMI): 25.1 ± 3.1 kg·m^−2^) were randomized to either a LC control group (LC-CON, *n* = 16), a LC and high-intensity interval training group (LC-HIIT, *n* = 17), or a LC and moderate-intensity continuous training group (LC-MICT, *n* = 17). All groups consumed LC for 4 weeks, while the LC-HIIT and LC-MICT groups followed an additional five sessions of HIIT (10 × 6 s cycling sprints and 9 s rest intervals, 2.5 min in total) or MICT (cycling continuously at 50–60% of peak oxygen uptake (VO_2peak_) for 30 min) weekly. Blood pressure, fasting glucose, insulin sensitivity, and several metabolic or appetite regulating hormones were measured before and after intervention. Results: Significant reductions in body weight (− ~2.5 kg, *p <* 0.001, *η^2^* = 0.772) and BMI (− ~1 unit, *p <* 0.001, *η^2^* = 0.782) were found in all groups. Systolic blood pressure was reduced by 5–6 mmHg (*p* < 0.001, *η^2^* = 0.370); fasting insulin, leptin, and ghrelin levels were also significantly decreased (*p <* 0.05), while insulin sensitivity was improved. However, there were no significant changes in fasting glucose, glucagon, and gastric inhibitory peptide levels. Furthermore, no group differences were found among the three groups, suggesting that extra training (i.e., LC-HIIT and LC-MICT) failed to trigger additional effects on these cardiometabolic profiles. Conclusions: The short-term carbohydrate restriction diet caused significant weight loss and improved blood pressure and insulin sensitivity in the overweight/obese women, although the combination with exercise training had no additional benefits on the examined cardiometabolic profiles. Moreover, the long-term safety and effectiveness of LC needs further study.

## 1. Introduction

Obesity is primarily due to the interaction between genetics and lifestyle factors, characterized by inappropriate nutrition and lack of regular physical activity [1]. It is closely accompanied by a series of metabolic abnormalities, including hyperglycemia, dyslipidemia, insulin deficiency, hypercoagulability, and altered fibrinolysis, causing sustained and progressive damage to blood vessel walls and insulin-sensitive tissues [1,2]. These abnormalities imply insulin resistance, inflammation, and endothelial dysfunction, which further contribute to the development of type 2 diabetes (T2D) and cardiovascular diseases (CVDs) [1,3]. Therefore, lifestyle interventions, such as diet management and exercise programs for overweight/obese people, are of great importance given the high prevalence of obesity-related metabolic diseases in the general population, which are beyond the administration of the hospital setting.

The low-carbohydrate diet (LC), a diet characterized by high-fat, adequate protein, and low levels of carbohydrates, is widely recognized as an effective strategy to manage obesity and improve glycemic control, insulin sensitivity, blood pressure, and lipidemic profiles [4,5,6,7,8,9,10,11]. We previously found that the overweight/obese Chinese females experienced marked reductions in body mass and abdominal fat mass after exposure to LC for 4 weeks [5]. In a well-controlled inpatient study, 14 days of LC administration normalized the plasma glucose concentrations, decreased hemoglobin A1c (HbA_1c_), and improved insulin sensitivity by approximately 75% in the obese T2D patients [6]. These short-term studies show that carbohydrate-restricted diets can have a rapid effect on cardiometabolic health. Furthermore, the LC-induced weight loss, improvements in HbA_1c_, and glycemic control could be retained for 22 months and even up to 44 months as revealed in follow-ups [10,11]. In fact, metabolic syndrome, insulin resistance, and T2D functionally manifest as “carbohydrate intolerance”. Insulin activates key enzymes in pathways that store energy from carbohydrates, and when dietary carbohydrate is restricted, the secretion of insulin is lowered, leading to reductions in lipogenesis and fat storage [4]. On the other hand, the body is compelled to seek alternative energy sources by boosting lipolysis and fat oxidation from fats stored in adipose tissue and supplied in diet [12]. Thus, reductions in fat and body mass and improvements in glucose, lipids, and insulin sensitivity occur as consequences of reduced carbohydrate supply and insulin secretion [13]. This is likely to be the mechanism via which LC diets reduce the cardiometabolic risk factors.

Despite the profound weight loss and cardiometabolic benefits, evidence suggests that LC exposure is associated with several adverse outcomes, including reduced cardiorespiratory fitness [14] and decreased muscle mass [15,16]. Under LC conditions, muscle glycogen stores and glycolytic-enzyme activities are reduced due to limited glucose availability, meanwhile, gluconeogenic activity is increased, which may have accelerated the breakdown of muscle mass and reduced cardiorespiratory fitness [17]. Interestingly, we previously found that when LC was combined with high intensity interval training (HIIT) or moderate intensity continuous training (MICT), the decline in cardiorespiratory fitness was reversed [7], and another study reported that the combination of LC and resistance training compensated for the muscle mass loss triggered by ketone bodies [18]. These findings suggest that additional exercise training can make up for the potential adverse effects of carbohydrate-restricted diets.

In addition to improving body composition and cardiorespiratory fitness, both HIIT and MICT are effective interventions to improve glucose regulation and insulin sensitivity, as well as blood pressure, with HIIT being more time efficient [19,20,21,22,23,24]. During exercise, more muscle fibers are recruited to participate in the activity, thus, speeding up the glucose disposal rate in active muscles [25], which in turn, promotes the action of insulin in assisting muscle glycogen resynthesis [26,27]. This is thought to be the first line mechanism by which exercise training leads to increased post-exercise glucose uptake and insulin action [28]. Since exercise and LC improve glucose regulation and insulin sensitivity through different metabolic pathways, it is reasonable to speculate that the combination of exercise training and LC may have synergistic effects on the beneficial health outcomes. However, evidence to date regarding the combined effects of LC and exercise training on cardiometabolic factors are not only limited but also inconsistent [7,16,18]. Therefore, it is our interest to further examine whether collaborating LC with time-saving HIIT or traditional MICT would trigger additional benefits on glucose regulation, insulin sensitivity, and blood pressure in overweight/obese individuals.

Given the above, this study was an effort to compare the effects of a 4-week LC intervention, with or without exercise training (i.e., HIIT or MICT), on fasting glucose, insulin sensitivity, and blood pressure in overweight/obese Chinese women and to examine whether the metabolic- or appetite-related hormones have played roles in the LC-induced regulation of cardiometabolic profiles. We hypothesized that the short-term LC intervention alone could improve certain cardiometabolic profiles, and additional benefits on glucose regulation, insulin sensitivity, or blood pressure could be derived when LC was combined with extra HIIT or MICT intervention.

## 2. Materials and Methods

### 2.1. Participants

**Figure 1:**
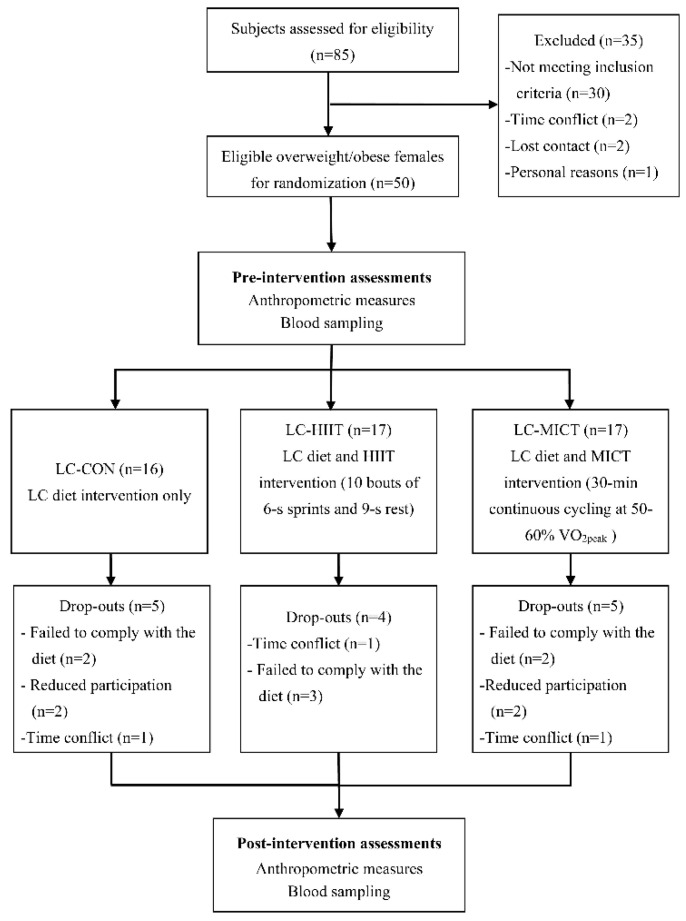
Flow-chart of the study.

This study was approved by the Research Ethics Committee of the University of Macau (RC Ref. no. MYRG2017-00199-FED) and conformed to the Declaration of Helsinki. Before recruitment, G * Power (Version 3.1) was used to calculate the sample size, the process of which was the same as that described in our previous study [7]. The power calculation results showed that 12 participants were sufficient for each group. With a potential dropout rate of 25%, we aimed to recruit a total of 45 participants for three groups. Participants who interested in this study were publicly recruited through advertising in the bulletin board of University of Macau and e-mail. The inclusion criteria were: (1) aged between 18–30 years old at enrolment, (2) overweight or obese defined as a body mass index (BMI) ≥23 kg/m^2^ [29], (3) body weight maintained stable in the past 6 months (±2 kg), (4) sedentary lifestyle (not participating in regular exercise or any structured exercise 6 months before enrollment), and (5) healthy (had not been diagnosed of any endocrine, metabolic, osteoarticular, or cardiovascular diseases and had no physical barriers to exercise). Participants were excluded if they were smokers, alcohol users, taking prescribed medicines or weight loss supplements, adhering to specific diet programs, participating in regular or structured exercise programs, or having respiratory problems or eating disorders. A total of 50 eligible overweight/obese women (19–25 years old) were screened out. After getting their written informed consents, participants were randomly assigned to one of three 4-week treatment groups: a low-carbohydrate diet control group (LC-CON, *n* = 16), a low-carbohydrate diet and HIIT group (LC-HIIT, *n* = 17), or a low-carbohydrate diet and MICT group (LC-MICT, *n* = 17). During the intervention, 14 participants dropped out for different reasons. Finally, 11, 13, and 12 participants in the LC-CON, LC-HIIT, and LC-MICT groups, respectively, who accomplished the whole intervention and all pre- and post-assessments were included in data analysis (Figure 1).

### 2.2. Study Design Overview

A pre- and post-test comparison design was used in this interventional study. The experimental procedure consisted of a preliminary stage, pre-intervention assessments of anthropometric data and blood assay, a 4-week intervention period, and post-intervention assessments (the flow-chart of the study is presented in Figure 1).

During the preliminary stage, all participants took nutrition workshops from a dietitian to learn and practice how to record all foods/drinks and quantities consumed, and how to choose the appropriate foods/drinks during LCs. Digital scales, standard food measuring utensils, and detailed instructions were given to all participants so that they could record the weight and amount of food/beverage intakes accurately. They were also instructed to record their baseline daily activities (in steps) and food intakes (normal diet) 3 days weekly (2 weekdays and 1 weekend day) on 2 weeks before intervention. Since data collection of all pre- and post-intervention measurements were performed at the same stage of each participant’s menstrual cycle (i.e., the luteal phase), we asked them to recall and report their menstrual cycles over the past 3 months in the preliminary stage. The time to measure outcome variables was then estimated based on their self-reported menstrual cycles. After the preliminary stage, participants completed the pre-intervention measures of anthropometric indices and blood collection 3–5 days before the intervention. During 4 weeks of intervention, participants in the LC-CON group served as a control group who changed from a normal diet to a LC diet but with no exercise training. In addition to the LC diet, participants in the LC-HIIT and LC-MICT groups followed five sessions of HIIT or MICT weekly in the lab. The post-intervention anthropometric measures and gathering of blood samples were carried out between 72–96 h after the last intervention day in the same way as the pre-intervention.

### 2.3. Diet Intervention

During the intervention, participants in LC-CON, LC-HIIT, and LC-MICT groups switched from a normal diet to a LC diet, in which approximately 65%, 25%, and 10% of their daily energy intakes were obtained from fats, proteins, and carbohydrates (~50 g/d), respectively. In addition to the nutrition workshops at the preliminary stage, all participants received personal dietary guidance from the dietitian on how to eat within the target nutrition requirements. Moreover, a handbook containing a food/drink list for LC and recipe examples, as well as “Points to Note on LC Diet”, was distributed to participants. They were free to choose low-carbohydrate foods according to their preferences but were asked not to change their daily energy intake as measured in the normal diet period (i.e., 2 weeks before intervention). Foods to choose from during LC included all kinds of fat, beef, cheese, eggs, fish, fowl (e.g., chickens, ducks), green vegetables, low-carbohydrate drinks (e.g., green/black tea, black coffee), non-starchy vegetables, nuts/seeds, oils, pork, seafoods, and water. No restriction was placed on the type of fat from saturated or unsaturated sources, and participants were encouraged to add five tablespoons of olive oil daily. Foods high in carbohydrates, including rice, noodles, bread, desserts, cereals, sweets, honey, beans, corns, starchy vegetables, fruits (except blueberries, lemons, and avocados), milk, yoghurt, fruit juices, soft drinks, and alcoholic beverages were avoided during the study period.

To ensure participants’ adherence to LC, we required them to take urinary ketone tests every day and record food logs 3 days/week (2 weekdays and 1 weekend day) during the study period. Participants measured their urinary ketones by themselves every morning or after dinner using the provided reagent strips (UROPAPER, Suzhou First Pharmaceutical Co. Ltd., Suzhou, China) [30] and recorded the results in the offered log book. The minimum detectable amount of the reagent strip was 10 mg/dL, and the concordance rate with clinical diagnosis was 95.1% [31]. Participants were asked to report urinary ketone test results and food records to the researchers on a weekly basis to assess dietary adherence and for further guidance. Energy intakes and macronutrient contents were analyzed by the same dietician using the nutrition analysis and management software (NRISM, version 3.1, China) according to the food records. Then, the personalized follow-up guidance and dietary recommendations were given to the participants based on these results. If participants had any questions, problems, or feedbacks about the study, they could contact the researchers through WeChat, phone, e-mail, or meet in person and get an immediate answer.

Apart from dietary and/or training intervention, participants were asked to maintain their habitual daily routines and not to participate in any additional exercises throughout the study period. Their daily physical activities were monitored using validated pedometers (Yamax Digi-Walker SW-200, Japan). Each participant received a logbook with calendar to record daily food intakes, physical activities (in steps), and urinary ketone test results, as well as adverse effects or symptoms relating to the intervention.

### 2.4. Exercise Intervention

In addition to switching from normal diet to LC diet, participants in the LC-HIIT and LC-MICT groups followed the 4-week prescribed exercise programs (five sessions/week) in the kinesiology lab, where the room temperature and humidity were strictly controlled at 22 °C and 50–60%, respectively. Two experienced research assistants supervised the whole training process. Participants in the LC-HIIT group were involved in a repeated sprint cycling exercise that included 10 repetitions of 6-s cycling sprints interspersed with 9-s passive recoveries (2.5 min/session in total) on a Monark ergometer (894E, Sweden). During the 6-s sprint periods, participants pedaled as fast as they could against an initial workload of 1 kg, whereas during the 9-s recovery periods, they rested on the seat. Once the participants were able to maintain a cycling speed of more than 100 rpm for all sprint bouts in two consecutive training sessions, the workload would be upgraded by 0.5 kg until the targeted workload was achieved (i.e., equivalent to 5% of their own body weight). Ratings of perceived exertion (RPE, Borg 6–20 scale) [32] were recorded before and immediately after the 5th and 10th exercise bouts. The power output and heart rate (HR) for each exercise bout were recorded automatically by the Monark Anaerobic Test software. Participants in the LC-MICT group performed continuous cycling exercise on a Monark ergometer (839E, Sweden) for 30 min. Maximal incremental exercise tests were carried out 2 days before intervention to calculate participant’s individual VO_2peak_ [5,7] and the workload used in MICT, which corresponded to 50% of VO_2peak_ for the first 10 sessions and increased to 60% of VO_2peak_ for the last 10 sessions. The pedaling speed was kept at 50 ± 5 rpm in all training sessions. RPE and HR were recorded in 5-min intervals.

To assess the energy expended during HIIT and MICT intervention, a gas analyzer (Vmax Encore, CareFusion Corp., San Diego, CA, USA) was used to measure oxygen uptake (VO_2_) during exercise breath-by-breath in the 1st, 10th, and 20th training sessions. The energy consumption for the exercise was calculated as 5.05 (Kcal·L^−1^) × VO_2_ (L·min^−1^) × exercise time (min), where VO_2_ was the average oxygen consumption value for the whole training session and the exercise time was 2.5 min for HIIT and 30 min for MICT.

### 2.5. Pre- and Post-Intervention Assessments

#### 2.5.1. Blood Analysis

Blood samples were taken by a qualified nurse before and 72 h after the last intervention. Strenuous exercise, caffeine, and alcohol were restricted 48 h before blood drawing. After 12 h of fasting, participants were required to arrive at the lab before 7:30 am to take a 5-mL blood sample from the cubital vein. After 1-h coagulation at room temperature (22 °C), serum was separated by centrifugation (3000 rpm for 5 min) and immediately stored at −80 °C for later analysis.

To minimize the inter-assay variation, blood tests were conducted at the end of the study by KingMed Diagnostics (Limited company, Guangzhou, China) in standard procedures according to the manufacturer’s instructions. Fasting glucose was assessed using an automatic biochemical analyzer (Olympus AU400, Olympus, Japan). Metabolic regulating hormones, including insulin, C-peptide, glucagon, leptin, ghrelin, and gastric inhibitory peptide (GIP), were measured using the EMD Millipore Milliplex MAP immunoassay (Merck KGaA, Darmstadt, Germany). Insulin sensitivity was assessed using the homeostasis model assessment of insulin resistance (HOMA-IR) index, calculating as: fasting serum insulin (μIU·mL^−1^) × fasting serum glucose (mmol·L^−1^)/22.5. The coefficients of variations for intra-assay and inter-assay were less than 3%.

#### 2.5.2. Resting Blood Pressure and Anthropometric Assessments

After blood sampling, blood pressure and anthropometric variables were assessed in the same morning. Blood pressure was measured twice on the right arm for each participant using an electronic sphygmomanometer (Microlife 3BTO-A, Taipei, Taiwan) after 10-min seating rest, when the difference between the two measurements was larger than 5 mmHg, the third measurement was taken, and the two nearest values were averaged and taken as systolic blood pressure (SBP) and diastolic blood pressure (DBP). Mean arterial pressure (MAP) was calculated as: (SBP + 2 × DBP)/3. Height and weight were measured via standard methods (without shoes and in light clothing) using a wall-mounted stadiometer and a digital scale, and the values were accurate to 0.1 cm and 0.1 kg, respectively. The BMI (in kg·m^−2^) was calculated as weight (kg) divided by height squared.

### 2.6. Statistical Analysis

Data were analyzed using the PASW software (Release 22.0; IBM, New York, NY, USA). Before performing the main statistical analyses, Shapiro–Wilk tests were conducted to confirm whether the outcome variables were normally distributed. Two-way repeated measures of ANOVA were conducted to determine the main effect (i.e., time effect) and interaction effects (time × group) on the main outcome variables. When significant interaction effects were observed, Tukey’s honestly significant difference (HSD) post hoc tests were performed to identify the difference among the three groups, in addition to the paired-sample t-tests to compare the main effect of time on outcome variables. Pearson’s correlation tests were used to evaluate the correlation between changes in cardiometabolic profiles and changes in metabolic-related hormones. To compare the differences in changes of outcome variables in response to the three interventions, one-way ANOVA tests were carried out, whilst independent samples T-test was used to detect differences in exercise training data between LC-HIIT and LC-MICT. Paired-sample t-tests were used to compare changes in dietary energy intakes, macronutrient compositions, and daily physical activities before (the mean value of 2 weeks at baseline) and during intervention (the mean value of 4 weeks of intervention). Partial *η^2^* values were used to assess effect sizes of the main and interaction effects, *η^2^* was considered small if <0.06 and large if >0.14 [33]. Cohen’s *d* values were also calculated to evaluate the effect sizes for the difference between variables, which was considered small when *d* was 0.2–0.3, medium when *d* was around 0.5, and large when *d* > 0.8 [34]. Data were presented as means (standard deviations, SDs), and the level of *p* < 0.05 was considered statistically significant.

## 3. Results

### 3.1. Training Data

For the two groups receiving extra training, the total training time spent in MICT (600 min) was as 12 times as the total training time spent in HIIT (50 min). Accordingly, the exercise energy expenditure of MICT (155.1 ± 16.4 kcal/session) was significantly larger than that of HIIT (18.4 ± 2.4 kcal/session, *p* < 0.01, Table 1). However, the exercise intensity of HIIT (86.5 ± 9.0% of VO_2peak_) was higher than that of MICT (63.2 ± 10.7% of VO_2peak_, *p* < 0.01). During exercise training, participants undertaking HIIT maintained a higher average HR (146 ± 5 bpm) than those involving in MICT (138 ± 8 bpm, *p* < 0.01). Additionally, HIIT (14 ± 1) was considered to be more strenuous than MICT (11 ± 1, *p* < 0.01) according to the self-reported RPE scores (Table 1).

### 3.2. Adverse Events

During the intervention, we received 18 feedbacks of adverse events from 14 participants in total, in which seven, six, and five complaints were from the LC-CON, LC-HIIT and LC-MICT groups, respectively. None of them were adverse clinical events. In contrast, no adverse feedback was reported during the 2-week normal diet period. Similar to the former study [14], fatigue (six or 33%), reduced appetite (four or 22%), constipation (five or 28%), diarrhea (two or 11%), and headache (one or 6%) were the main adverse events reported by the participants.

### 3.3. Diet Compliance, Dietary Compositions and Daily Physical Activities

Participants were able to detect urinary ketones after eating LC diet for about 3 days. After excluding the data from the first 3 days, positive urinary ketoses were detected on 95.6 ± 5.0%, 96.6 ± 5.3%, and 96.7 ± 6.2% of the days in the LC-CON, LC-HIIT, and LC-MICT groups, respectively. This indicated that participants had good compliance to LC. In comparison, urinary ketosis was only detected on 1.1 ± 2.7% of the days during the 2-week normal diet period.

At baseline, there was no difference in daily energy intake and macronutrient compositions in the three groups. The mean daily energy intake of normal diet was 2071 ± 407 kcal, in which 44.7 ± 8.3%, 15.0 ± 2.3%, and 37.1 ± 7.0% of the total energy intake was derived from carbohydrates (241 ± 58 g), proteins (79 ± 19 g), and fats (89 ± 22 g). When changed to LC diet, the daily energy intake of each group had no significant change compared with the normal diet (*p* > 0.05), which were 1857 ± 282 kcal, 1828 ± 204 kcal, and 1984 ± 254 kcal in LC-CON, LC-HIIT, and LC-MICT, respectively. However, the proportion of macronutrients in the daily energy intake changed significantly in all groups, with higher intake of protein (111 ± 25 g in LC vs. 79 ± 19 g in normal diet, *p* < 0.01) and fat (141 ± 18 g in LC vs. 88 ± 22 g in normal diet, *p* < 0.01) and lower intake of carbohydrate (46 ± 15 g in LC vs. 241 ± 58 g in normal diet, *p* < 0.01) as compared to that of normal diet. The proportions of energy intake derived from proteins and fats during LC diet in the three groups were increased to approximate 23.5% and 67.0%, whereas the proportion of carbohydrates decreased to 9.9% (data of changes in diet before and during intervention are shown in Table 2).

There were no statistical differences in daily physical activities before and during the intervention in the LC-CON group (7405 ± 1629 steps in LC vs. 8003 ± 852 steps in normal diet, *p >* 0.05), the LC-HIIT group (8254 ± 1377 steps in LC vs. 7749 ± 1684 steps in normal diet, *p >* 0.05), and the LC-MICT group (8399 ± 1186 steps in LC vs. 8194 ± 1761 steps in normal diet, *p >* 0.05) (data are presented in Supplementary Materials Table S1).

### 3.4. Results of Main Outcomes

After intervention, participants in all groups reduced body weight (*p <* 0.01, *η^2^* = 0.772) and BMI (*p <* 0.01, *η^2^* = 0.782) significantly, with no group differences (Table 3).

Specifically, the LC-CON, LC-HIIT, and LC-MICT groups reduced body weight by 2.5 ± 1.8, 2.7 ± 1.3, and 2.4 ± 1.3 kg and lowered BMI by 0.9 ± 0.6, 1.0 ± 0.5, and 0.9 ± 0.5 kg/m^2^, respectively (Table 4). The three interventions also triggered similar reductions in SBP (*p <* 0.01, *η^2^* = 0.370) and MAP (*p <* 0.01, *η^2^* = 0.321). In terms of blood profiles, circulating concentrations of insulin (*p <* 0.01, *η^2^* = 0.481), leptin (*p <* 0.01, *η^2^* = 0.342), and ghrelin (*p <* 0.01, *η^2^* = 0.244) were significantly decreased after intervention in all groups, accompanied with improved insulin sensitivity, as reflected by reductions in HOMA-IR index (*p <* 0.01, *η^2^* = 0.456). However, there were no statistical differences on fasting glucose, as well as the fasting C-peptide, glucagon, and GIP concentrations after 4 weeks of intervention (*p >* 0.05, Table 3).

To our surprise, there were no correlations between changes in body weight, BMI, blood pressure, fasting glucose, and the changes in metabolic-related hormones (i.e., insulin, glucagon, C-peptide, leptin, and ghrelin) (*p >* 0.05). In contrast, changes in insulin level were positively correlated with changes in C-peptide (*r* = 0.337, *p <* 0.05) and leptin (*r* = 0.372, *p* < 0.05) levels, and changes in C-peptide and leptin were correlated with each other as well (*r* = 0.660, *p* < 0.01).

## 4. Discussion

In our previous study, we demonstrated that short-term LC intervention reduced body weight and visceral fat mass in overweight/obese women effectively [5,7]. This study further demonstrated that, in addition to weight loss, 4 weeks of LC intervention lowered blood pressure, improved insulin sensitivity, and reduced hormone levels of insulin, leptin, and ghrelin in the overweight/obese Chinese young women, which are factors known to decrease CVD and T2D risks. However, contrary to our hypothesis, a combination with extra training (i.e., HIIT and MICT) had no additional benefits on any of the examined cardiometabolic factors, although previous studies by us and others have found extra effects of exercise training in improving cardiorespiratory fitness [7] and maintaining muscle mass [18].

The overweight/obese Chinese women lost approximately 2.5 kg of body weight (or roughly 1 unit of BMI) in response to 4 weeks of LC administration. This finding was in support of our previous studies [5,7]. Without changing dietary energy intake, the noted weight loss in this study can be ascribed to carbohydrate restriction per se. Similar results were found in both LC-HIIT and LC-MICT groups, suggesting that there was no collaborative effect of exercise training on weight loss. Consistent with previous studies in overweight or obese women [8,9], LC intervention with or without exercise training yielded similar reductions in SBP (−5–6 mmHg) and DBP (−2–4 mmHg) in this study, although most of the overweight females started out with a normal blood pressure. Such a decline is notable because blood pressure, especially SBP, is a recognized independent predictor of fatal CVD, even for individuals within the normotensive range [35]. Every 10 mmHg reduction in SBP can lead to a significant 13% reduction in CVD risks and all-cause mortality [36]. Therefore, the 5–6 mmHg reduction in SBP is of clinical significance in terms of reducing CVD risks.

Insulin resistance is a precursor to a series of obesity-related diseases and always closely coexist with T2D [1]. Both carbohydrate-restricted diet [6,10,11,14] and exercise [22,23,28] have been consistently reported to improve glycemic control and insulin sensitivity effectively. Although fasting blood glucose was unchanged in this study, there was a significant reduction in post-LC insulin levels, accompanied with improved insulin sensitivity as indicated by the HOMA-IR index. In contrast to our hypothesis, the combined diet and exercise did not add benefits on glucose regulation or insulin sensitivity compared to diet alone. The benefit of exercise training in improving insulin sensitivity is essentially due to the depletion of muscle glycogen [26,27], yet, in the LC condition, muscle glycogen depletion seemed to be achieved solely by limiting exogenous carbohydrate intake. The addition of HIIT and MICT did not show a synergistic effect, suggesting that carbohydrate restriction may have already maximized this mechanism. Moreover, exercise-induced energy deficit is also thought to play an important role in mediating the beneficial effects of exercise on systemic and hepatic insulin sensitivity [28]. Compared to LC-CON, LC-HIIT and LC-MICT caused an energy deficit of ~400 kcal and ~3000 kcal respectively in the present study. However, no additional benefits in the examined cardiometabolic parameters were observed in these groups. This observation reinforced the notion that the improvement in insulin sensitivity depends more on reducing exogenous glucose supply (or carbohydrate availability) rather than on energy deficits [37]. The practical implication of these findings is that occasionally missing exercise can substitute for some exercise-induced health benefits (e.g., improving insulin sensitivity) by limiting carbohydrate intake.

The mechanisms involved in the LC-induced weight loss and fat loss have not been definitively established but could be regulated (at least in part) by changes in peripheral appetite and metabolic modulators [38,39]. To verify this potential mechanism, this study examined several hormones known to affect appetite and substance metabolism, which include insulin, glucagon, leptin, ghrelin, and GIP. We found that serum levels of glucagon and GIP were unaffected after the LC intervention, regardless of the presence or absence of additional exercise. Interestingly, not all LC-sensitive hormones (i.e., insulin, leptin, and ghrelin) in the present study switched in a direction to suppress appetite. Leptin is a hormone produced by adipose tissue and positively associated with fat stores that reduces food intake while increasing energy expenditure [40]. However, we found a declined leptin level after 4 weeks of LC consumption, which was in line with many previous studies [6,38,39]. It has been reported that plasma leptin level during dynamic weight loss was lower than that during weight loss maintenance, suggesting that leptin acts more as an emergency signal of energy depletion than an inhibitor of food intake [41]. Furthermore, we found no correlation between the changes of body weight and leptin. Therefore, it is more likely that the lower leptin levels in this study are hormonal compensatory changes that encourage energy balance restoration and weight regain [39,41].

Both insulin and ghrelin are recognized as orexigenic hormones. Insulin promotes substrate storage by stimulating lipogenesis and inhibiting lipolysis [42], while ghrelin causes weight gain by reducing fat utilization [43]. Unlike the consistency in the insulin reduction results after ingesting LC diets [6,10,11,14], studies on the response of ghrelin to LC exposure yielded conflicting findings, with reports of either unchanged [38,39] or increased levels [6]. In the current study, both of the orexigenic hormone levels (i.e., insulin and ghrelin) were decreased, though they did not appear to play a role in LC-induced weight loss, considering that changes in body weight were not associated with changes in circulating insulin or ghrelin concentrations at the overall level. In addition, the participants did not change their daily energy intake throughout the study, indicating an unchanged appetite. Nonetheless, we acknowledged that measuring circulating hormone concentrations alone does not necessarily reflect changes in hormone biosynthesis/secretion, signal induction, or receptor uptake. To clarify the mechanism by which LC induces weight loss, further examination of more sophisticated molecular biomarkers is needed, and other appetite or metabolism regulating hormones (e.g., thyroid hormones, cortisol, epinephrine, growth hormone) should be involved.

Of note, the current study has several limitations. First, given that our previous studies have examined the effects of LC diet on body composition [5,7], this was not the main research scope of this study. However, lacking sophisticated body composition variables has somewhat limited the interpretation of hormone results in the current study. Second, none of the biosynthesis/secretion, signal induction, or receptor uptake of the related hormones was measured in this study, which has also limited the in-depth understanding of the findings. Third, since standard pre-prepared LC meals were not provided, participants selected low-carbohydrate foods/beverages by themselves according to the nutritional instructions, and food intake was also self-reported; therefore, we cannot rule out the possibility of underreporting or overreporting. Future studies could consider more strict control of macronutrient proportions by providing standard LC meals. Additionally, although our short-term LC trial has demonstrated favorable cardiometabolic outcomes, the long-term safety of LC deserves further evaluation, as evidence shows that long-term consumption of LC increases the risks of higher low-density lipoprotein cholesterol (LDL-C) levels [44], osteoporosis [45], and all-cause mortality [46]. Continuous consumption of higher dietary fat during LCs has raised concerns about the influence of LCs on blood lipids, especially the LDL-C, which is a recognized predictor for major cardiovascular events [47]. In spite of favorable changes in triglyceride and high-density lipoprotein cholesterol, several studies have linked LCs to elevated LDL-C levels [44]. Therefore, periodic testing of blood lipids is necessary while performing LCs.

## 5. Conclusions

In summary, the present study demonstrated that the short-term (i.e., 4-week) LC diet was able to elicit marked weight loss; improve blood pressure and insulin sensitivity; and reduce fasting insulin, leptin, and ghrelin levels in overweight/obese but otherwise healthy women. However, the addition of HIIT or MICT to the carbohydrate restriction diet did not enhance the improvements in any of the examined cardiometabolic profiles. Although favorable changes in cardiometabolic health were observed in this short-term study, these findings should be interpreted with caution when extrapolating the long-term effects of LC from the short-term study results. Further studies are expected to reveal the long-term safety and effectiveness of LC on cardiometabolic profiles and how long these effects can last.

## Figures and Tables

**Table 1:** Training variables during exercise intervention.

	KD-HIIT	KD-MICT
Weekly training time (min)	12.5	150
Total training time (min)	50	600
Energy expenditure (kcal)	18.4 (2.4) **	155.1 (16.4)
Training power (W)	242.1 (24.3) **	53.2 (9.8)
Intensity (%VO_2peak_)	86.5 (9.0) **	63.2 (10.7)
Training HR (bpm)	146 (5) **	138 (8)
Training HR/HR_max_ (%)	82.1 (3.9) **	74.4 (2.6)
Training RPE	14 (1) **	11 (1)

All values are presented as means (standard deviations). LC-HIIT: low-carbohydrate diet combined with high-intensity interval training, LC-MICT: low-carbohydrate diet combined with moderate-intensity continuous training. Comparison with LC-MICT at ** *p* < 0.01.

**Table 2:** Dietary energy intake and macronutrient compositions before and during intervention.

	Pre Week 1	Pre Week 2	Week 1	Week 2	Week 3	Week 4
Energy intake (kcal/d)						
LC-CON	2038 (409.6)	2069 (317.2)	1917 (426.8)	1756 (564.5)	1823 (495.0)	1908 (296.0)
LC-HIIT	2066 (362.9)	2158 (312.3)	1877 (381.0)	1868 (274.2)	1861 (358.2)	1942 (420.5)
LC-MICT	2175 (411.3)	1968 (535.8)	2041 (437.6)	2031 (536.8)	1930 (456.2)	1913 (440.6)
Carbohydrate (in %)						
LC-CON	44.6 (8.1)	40.6 (11.7)	10.4 (4.3)	9.0 (3.6)	7.7 (2.9)	8.1 (5.6)
LC-HIIT	48.4 (7.5)	44.9 (10.0)	13.5 (8.0)	11.7 (5.5)	8.7 (5.4)	7.4 (2.9)
LC-MICT	44.8 (9.5)	45.7 (10.7)	13.1 (8.3)	10.0 (4.2)	9.1 (2.9)	8.8 (3.0)
Carbohydrate (in g)						
LC-CON	239.6 (80.8)	208.6 (60.6)	51.6 (26.8)	38.3 (14.5)	34.4 (12.9)	37.8 (23.5)
LC-HIIT	278.6 (100.3)	242.9 (64.9)	55.6 (31.5)	55.1 (27.4)	43.2 (35.0)	34.2 (13.9)
LC-MICT	241.0 (60.7)	227.4 (88.3)	62.9 (41.4)	52.6 (29.6)	43.6 (15.4)	42.4 (18.3)
Fat (in %)						
LC-CON	38.8 (8.1)	38.9 (9.7)	68.8 (6.8)	67.2 (5.2)	68.5 (5.3)	69.9 (5.8)
LC-HIIT	34.8 (5.4)	37.1 (7.9)	64.6 (8.1)	63.8 (8.8)	69.6 (7.5)	68.8 (10.5)
LC-MICT	37.4 (8.9)	35.3 (8.8)	63.3 (8.6)	64.5 (7.3)	67.8 (6.1)	68.0 (7.3)
Fat (in g)						
LC-CON	88.3 (14.8)	95.0 (27.8)	146.7 (36.5)	131.6 (44.4)	139.9 (44.4)	149.0 (31.4)
LC-HIIT	87.0 (25.1)	88.8 (21.2)	123.6 (21.2)	131.7 (22.7)	143.0 (26.9)	141.3 (39.2)
LC-MICT	91.4 (31.4)	75.6 (22.1)	145.6 (42.9)	145.8 (41.4)	145.6 (35.8)	143.4 (29.8)
Protein (in %)						
LC-CON	15.9 (4.5)	15.3 (2.2)	22.1 (5.5)	24.3 (4.9)	24.3 (4.7)	22.1 (4.6)
LC-HIIT	14.8 (2.1)	15.4 (3.1)	23.2 (5.6)	23.8 (7.2)	21.8 (4.7)	23.6 (8.4)
LC-MICT	13.7 (2.3)	14.8 (2.6)	23.4 (5.3)	24.9 (4.6)	23.2 (5.3)	23.3 (5.5)
Protein (in g)						
LC-CON	79.4 (23.2)	81.3 (18.5)	104.5 (31.6)	106.6 (39.7)	108.8 (27.2)	104.6 (24.5)
LC-HIIT	83.4 (22.3)	82.5 (18.8)	100.1 (27.8)	112.7 (41.2)	101.0 (25.4)	112.2 (49.8)
LC-MICT	74.1 (19.0)	72.2 (21.1)	118.7 (30.7)	127.8 (45.4)	112.0 (37.5)	113.8 (46.3)

Outcome variables are presented as mean (standard deviation). LC-CON: low-carbohydrate diet control group; LC-HIIT: low-carbohydrate diet and high-intensity interval training; LC-MICT: low-carbohydrate diet and moderate-intensity continuous training.

**Table 3:** Outcome variables before and after intervention.

	LC-CON (*n* = 11)		LC-HIIT (*n* = 13)		LC-MICT (*n* = 12)		Time Effect		Interaction Effect (Time × Group)	
	Pre	Post	Pre	Post	Pre	Post	*p*	*η^2^*	*p*	*η^2^*
Age (y)	21.6 (4.3)		21.4 (2.9)		21.8 (3.1)					
Height (cm)	161.4 (4.1)		163.5 (6.4)		161.1 (4.4)					
Weight (kg)	64.6 (9.3)	62.1 (8.3) ^	66.7 (8.8)	64.0 (8.3) ^	64.4 (6.7)	61.9 (6.3) ^	0.000	0.772	0.902	0.006
BMI (kg·m^−2^)	24.8 (3.3)	23.8 (3.1) ^	24.8 (2.0)	23.9 (1.9) ^	24.8 (1.9)	23.8 (1.9) ^	0.000	0.782	0.946	0.003
SBP (mmHg)	113 (11)	108 (9) *	111 (8)	106 (13) *	111 (8)	106 (10) *	0.000	0.370	0.973	0.002
DBP (mmHg)	71 (9)	69 (8)	70 (6)	66 (8) *	71 (5)	68 (9)	0.005	0.212	0.564	0.034
MAP (mmHg)	85 (9)	82 (8)	84 (6)	79 (9) *	84 (5)	80 (9) *	0.000	0.321	0.811	0.013
FG (mmol·L^−1^)	4.8 (0.3)	4.6 (0.5)	4.8 (0.5)	4.7 (0.6)	4.8 (0.3)	4.7 (0.3)	0.071	0.095	0.928	0.004
Insulin (µIU·mL^−1^)	14.0 (5.8)	9.3 (6.2) *	13.3 (7.4)	9.4 (5.8) ^	14.4 (9.0)	11.3 (6.5) *	0.000	0.481	0.663	0.025
C-peptide (pg·mL^−1^)	1016.7 (368.9)	911.7 (440.2)	1218.8 (470.8)	968.0 (335.6)	1072.9 (336.9)	801.8 (343.2)	0.015	0.171	0.672	0.025
HOMA-IR	3.0 (1.3)	2.0 (1.5) *	2.9 (1.7)	2.0 (1.3) ^	3.2 (2.1)	2.4 (1.5) *	0.000	0.456	0.811	0.013
Glucagon (pg·mL^−1^)	64.7 (33.1)	64.6 (33.5)	49.2 (16.8)	62.4 (27.3)	51.4 (17.5)	61.6 (26.2)	0.244	0.045	0.706	0.023
Leptin (ng·mL^−1^)	13.5 (9.5)	7.6 (7.7) *	11.4 (5.4)	6.6 (3.6) *	15.1 (7.0)	7.5 (6.9) *	0.000	0.342	0.738	0.019
Ghrelin (pg·mL^−1^)	860.7 (704.3)	698.4 (584.2)	588.5 (324.9)	517.1 (231.7)	794.1 (400.1)	619.8 (463.4) *	0.003	0.244	0.555	0.036
GIP (pg·mL^−1^)	45.2 (15.7)	63.0 (35.9)	43.2 (11.2)	57.4 (33.4)	49.5 (27.6)	53.1 (26.5)	0.062	0.104	0.624	0.029

Main outcomes are shown as mean (standard deviations). Within subjects comparison from pre- and post-measures at * *p* < 0.05, ^ *p* < 0.01; Partial *η^2^* value for effect size (ES). LC-CON: low-carbohydrate diet control group; LC-HIIT: low-carbohydrate diet and high-intensity interval training; LC-MICT: low-carbohydrate diet and moderate-intensity continuous training; BMI: body mass index; SBP: systolic blood pressure; DBP: diastolic blood pressure; MAP: mean arterial pressure; FG: fasting glucose; HOMA-IR: homeostasis model assessment of insulin resistance; GIP: gastric inhibitory peptide.

**Table 4:** Changes in outcome variables after intervention.

	∆ LC-CON	∆ LC-HIIT	∆ LC-MICT	ES (d)	
	(*n* = 11)	(*n* = 13)	(*n* = 12)	KD-HIIT	KD-MICT
∆ Weight (kg)	−2.5 (1.8)	−2.7 (1.3)	−2.4 (1.3)	0.12	0.04
∆ BMI (kg·m^−2^)	−0.9 (0.6)	−1.0 (0.5)	−0.9 (0.5)	0.10	0.03
∆ SBP (mmHg)	−5 (6)	−5 (8)	−6 (7)	0.00	0.09
∆ DBP (mmHg)	−2 (7)	−4 (6)	−3 (6)	0.42	0.19
∆ MAP (mmHg)	−3 (6)	−5 (6)	−4 (5)	0.26	0.16
∆ FG (mmol·L^−1^)	−0.2 (0.6)	−0.2 (0.5)	−0.1 (0.3)	0.01	0.14
∆ Insulin (µIU·mL^−1^)	−4.8 (5.4)	−3.9 (3.0)	−3.2 (4.3)	0.20	0.34
∆ HOMA-IR	−1.0 (1.3)	−0.8 (0.7)	−0.7 (1.0)	0.20	0.26
∆ Glucagon (pg·mL^−1^)	−0.1 (40.5)	13.2 (35.5)	10.2 (36.4)	0.35	0.27
∆ C-peptide (pg·mL^−1^)	−105.0 (412.6)	−250.8 (519.8)	−271.1 (496.4)	0.31	0.36
∆ HOMA-IR	−1.0 (1.3)	−0.8 (0.7)	−0.7 (1.0)	0.20	0.26
∆ Glucagon (pg·mL^−1^)	−0.1 (40.5)	13.2 (35.5)	10.2 (36.4)	0.35	0.27
∆ Leptin (ng·mL^−1^)	−5.9 (8.2)	−4.8 (7.5)	−7.6 (10.3)	0.15	0.18
∆ Ghrelin (pg·mL^−1^)	−162.3 (325.4)	−71.4 (161.9)	−174.3 (244.6)	0.36	0.04
∆ GIP (pg·mL^−1^)	17.8 (34.5)	14.1 (25.5)	3.6 (45.9)	0.12	0.35

Outcome variables are presented as mean (standard deviation). Delta (∆): change from pre- to post-intervention; LC-CON: low-carbohydrate diet control group; LC-HIIT: low-carbohydrate diet and high-intensity interval training; LC-MICT: low-carbohydrate diet and moderate-intensity continuous training; BMI: body mass index; SBP: systolic blood pressure; DBP: diastolic blood pressure; MAP: mean arterial pressure; FG: fasting glucose; HOMA-IR: homeostasis model assessment of insulin resistance; GIP: gastric inhibitory peptide.

## Data Availability

The data presented in this study are available on request from the corresponding author. The data are not publicly available due to the wishes of some subjects.

## Supplementary Materials

The following are available online at https://www.mdpi.com/article/10.3390/healthcare9060637/s1, Table S1: Daily physical activities before and during intervention.

## References

[1] Caballero A.E. (2003). Endothelial Dysfunction in Obesity and Insulin Resistance: A Road to Diabetes and Heart Disease. Obes. Res..

[2] Olokoba A.B., Obateru O.A., Olokoba L.B. (2012). Type 2 Diabetes Mellitus: A Review of Current Trends. Oman Med. J..

[3] Gómez-Ambrosi J., Catalán V., Rodríguez A., Andrada P., Ramírez B., Ibáñez P., Vila N., Romero S., Margall M.A., Gil M.J. (2014). Increased Cardiometabolic Risk Factors and Inflammation in Adipose Tissue in Obese Subjects Classified as Metabolically Healthy. Diabetes Care.

[4] Paoli A., Rubini A., Volek J.S., Grimaldi K. (2013). Beyond weight loss: A review of the therapeutic uses of very-low-carbohydrate (ketogenic) diets. Eur. J. Clin. Nutr..

[5] Kong Z., Sun S., Shi Q., Zhang H., Tong T.K., Nie J. (2020). Short-Term Ketogenic Diet Improves Abdominal Obesity in Overweight/Obese Chinese Young Females. Front. Physiol..

[6] Boden G., Sargrad K., Homko C., Mozzoli M., Stein T.P. (2005). Effect of a Low-Carbohydrate Diet on Appetite, Blood Glucose Levels, and Insulin Resistance in Obese Patients with Type 2 Diabetes. Ann. Intern. Med..

[7] Sun S., Kong Z., Shi Q., Hu M., Zhang H., Zhang D., Nie J. (2019). Non-Energy-Restricted Low-Carbohydrate Diet Combined with Exercise Intervention Improved Cardiometabolic Health in Overweight Chinese Females. Nutrients.

[8] Liu X., Zhang G., Ye X., Li H., Chen X., Tang L., Feng Y., Shai I., Stampfer M.J., Hu F.B. (2013). Effects of a low-carbohydrate diet on weight loss and cardiometabolic profile in Chinese women: A randomised controlled feeding trial. Br. J. Nutr..

[9] Meckling K.A., Gauthier M., Grubb R., Sanford J. (2002). Effects of a hypocaloric, low-carbohydrate diet on weight loss, blood lipids, blood pressure, glucose tolerance, and body composition in free-living overweight women. Can. J. Physiol. Pharmacol..

[10] Nielsen J.V., Joensson E. (2006). Low-carbohydrate diet in type 2 diabetes. Stable improvement of bodyweight and glycemic control during 22 months follow-up. Nutr. Metab..

[11] Nielsen J.V., Joensson E. (2008). Low-carbohydrate diet in type 2 diabetes: Stable improvement of bodyweight and glycemic control during 44 months follow-up. Nutr. Metab..

[12] Paoli A., Bianco A., Grimaldi K.A. (2015). The ketogenic diet and sport: A possible marriage?. Exerc. Sport Sci. Rev..

[13] Ludwig D.S., Friedman M.I. (2014). Increasing adiposity: Consequence or cause of overeating?. JAMA.

[14] Urbain P., Strom L., Morawski L., Wehrle A., Deibert P., Bertz H. (2017). Impact of a 6-week non-energy-restricted ketogenic diet on physical fitness, body composition and biochemical parameters in healthy adults. Nutr. Metab..

[15] Tinsley G.M., Willoughby D.S. (2016). Fat-Free Mass Changes During Ketogenic Diets and the Potential Role of Resistance Training. Int. J. Sport Nutr. Exerc. Metab..

[16] Perissiou M., Borkoles E., Kobayashi K., Polman R. (2020). The Effect of an 8 Week Prescribed Exercise and Low-Carbohydrate Diet on Cardiorespiratory Fitness, Body Composition and Cardiometabolic Risk Factors in Obese Individuals: A Randomised Controlled Trial. Nutrients.

[17] Soeters M.R., Soeters P.B., Schooneman M.G., Houten S., Romijn J.A. (2012). Adaptive reciprocity of lipid and glucose metabolism in human short-term starvation. Am. J. Physiol. Metab..

[18] Jabekk P.T., Moe I.A., Meen H.D., Tomten S.E., Høstmark A.T. (2010). Resistance training in overweight women on a ketogenic diet conserved lean body mass while reducing body fat. Nutr. Metab..

[19] Sun S., Zhang H., Kong Z., Shi Q., Tong T.K., Nie J. (2018). Twelve weeks of low volume sprint interval training improves cardio-metabolic health outcomes in overweight females. J. Sports Sci..

[20] Trapp E.G., Chisholm D.J., Freund J., Boutcher S.H. (2008). The effects of high-intensity intermittent exercise training on fat loss and fasting insulin levels of young women. Int. J. Obes..

[21] Martins C., Kazakova I., Ludviksen M., Mehus I., Wisloff U., Kulseng B., Morgan L., King N. (2016). High-Intensity Interval Training and Isocaloric Moderate-Intensity Continuous Training Result in Similar Improvements in Body Composition and Fitness in Obese Individuals. Int. J. Sport Nutr. Exerc. Metab..

[22] Kong Z., Shi Q., Sun S., Tong T.K., Zhang H., Nie J. (2019). High-intensity interval exercise lowers postprandial glucose concentrations more in obese adults than lean adults. Prim. Care Diabetes.

[23] Nie J., Kong Z., Baker J.S., Tong T.K., Lei S.H., Shi Q. (2012). Acute changes in glycemic homeostasis in response to brief high-intensity intermittent exercise in obese adults. J. Exerc. Sci. Fit..

[24] Caldas Costa E., Hay J.L., Kehler D.S., Boreskie K.F., Arora R.C., Umpierre D., Szwajcer A., Duhamel T.A. (2018). Effects of high-intensity interval training versus moderate-intensity continuous training on blood pressure in prehypertensive and hypertensive individuals: A systematic review and meta-analysis of randomized trials. Sports Med..

[25] Wright D.C., Swan P.D. (2001). Optimal Exercise Intensity for Individuals with Impaired Glucose Tolerance. Diabetes Spectr..

[26] Bogardus C., Thuillez P., Ravussin E., Vasquez B., Narimiga M., Azhar S. (1983). Effect of muscle glycogen depletion on in vivo insulin action in man. J. Clin. Investig..

[27] Devlin J.T., Hirshman M., Horton E.D., Horton E.S. (1987). Enhanced peripheral and splanchnic insulin sensitivity in NIDDM men after single bout of exercise. Diabetes.

[28] Black S.E., Mitchell E., Freedson P.S., Chipkin S.R., Braun B. (2005). Improved insulin action following short-term exercise training: Role of energy and carbohydrate balance. J. Appl. Physiol..

[29] World Health Organization (2000). The Asia-Pacific Perspective: Redefining Obesity and Its Treatment.

[30] Urbain P., Bertz H. (2016). Monitoring for compliance with a ketogenic diet: What is the best time of day to test for urinary ketosis?. Nutr. Metab..

[31] Ishizaki D., Uchimoto T., Saito M., Sawabe Y., Matsushita K. (2016). Comparison of three automated urine analyzer models in terms of their basic performance and abnormal reaction detection system. Jpn. J. Med. Technol..

[32] Borg G. (1977). Simple rating methods for estimation of perceived exertion. Phys. Work Effort.

[33] Levine T.R., Hullett C.R. (2010). Eta Squared, Partial Eta Squared, and Misreporting of Effect Size in Communication Research. Hum. Commun. Res..

[34] Cohen J. (2013). Statistical Power Analysis for the Behavioral Sciences.

[35] Pei D., Chen Y.-L., Tang S.-H., Wu C.-Z., Lin J.-D., Chang Y.-L., Hsu C.-H., Wang C.-Y., Wang K., Wang J.-Y. (2011). Relationship of Blood Pressure and Cardiovascular Disease Risk Factors in Normotensive Middle-Aged Men. Medicine.

[36] Ettehad D., Emdin A.C., Kiran A., Anderson S.G., Callender T., Emberson J., Chalmers J., Rodgers A., Rahimi K. (2016). Blood pressure lowering for prevention of cardiovascular disease and death: A systematic review and meta-analysis. Lancet.

[37] Harry T., Wu C.L., Chen Y.C., Wang P.G., Javier G., James B. (2018). Post-Exercise Carbohydrate-Energy Replacement Attenuates Insulin Sensitivity and Glucose Tolerance the Following Morning in Healthy Adults. Nutrients.

[38] Ratliff J., Mutungi G., Puglisi M.J., Volek J.S., Fernandez M.L. (2009). Carbohydrate restriction (with or without additional dietary cholesterol provided by eggs) reduces insulin resistance and plasma leptin without modifying appetite hormones in adult men. Nutr. Res..

[39] Sumithran P., Prendergast L.A., Delbridge E., Purcell K., Shulkes A., Kriketos A., Proietto J. (2013). Ketosis and appetite-mediating nutrients and hormones after weight loss. Eur. J. Clin. Nutr..

[40] Ahima R.S., Flier J.S. (2000). Leptin. Annu. Rev. Physiol..

[41] Rosenbaum M., Nicolson M., Hirsch J., Murphy E., Chu F., Leibel R.L. (1997). Effects of Weight Change on Plasma Leptin Concentrations and Energy Expenditure1. J. Clin. Endocrinol. Metab..

[42] Saltiel A.R., Kahn C.R. (2001). Insulin signalling and the regulation of glucose and lipid metabolism. Nat. Cell Biol..

[43] Tschop M.H., Smiley D.L., Heiman M.L. (2000). Ghrelin induces adiposity in rodents. Nat. Cell Biol..

[44] Bueno N.B., de Melo I.S.V., de Oliveira S.L., da Rocha Ataide T. (2013). Very-low-carbohydrate ketogenic diet v. low-fat diet for long-term weight loss: A meta-analysis of randomised controlled trials. Br. J. Nutr..

[45] Bergqvist A.G.C., Schall J.I., Stallings V.A., Zemel B.S. (2008). Progressive bone mineral content loss in children with intractable epilepsy treated with the ketogenic diet. Am. J. Clin. Nutr..

[46] Hiroshi N., Atsushi G., Tetsuro T., Noda M. (2013). Low-Carbohydrate Diets and All-Cause Mortality: A Systematic Review and Meta-Analysis of Observational Studies. PLoS ONE.

[47] Barter P., Gotto A.M., LaRosa J.C., Maroni J., Szarek M., Grundy S.M., Kastelein J.J.P., Bittner V., Fruchart J.C. (2007). HDL Cholesterol, Very Low Levels of LDL Cholesterol, and Cardiovascular Events. N. Engl. J. Med..

