# Supplementary material for: Carbohydrate Restriction with or without Exercise Training Improves Blood Pressure and Insulin Sensitivity in Overweight Women
Source: Healthcare (Basel). 2021 May 27;9(6):637. doi: 10.3390/healthcare9060637 (PMC8229341; doi:10.3390/healthcare9060637)
Supplement: Supplementary file 1 [file healthcare-09-00637-s001.zip › healthcare-1221677-supplementary.pdf]

Table S1 Daily physical activities before and during intervention

Steps	Pre_week 1	Pre_week 2	Week 1	Week 2	Week 3	Week 4
LC-CON	8180 (1686)	7624 (1719)	7314 (2064)	7958 (3036)	7425 (2802)	7696 (1166)
LC-HIIT	7997 (3446)	7501 (2794)	8231 (1930)	8213 (2166)	8734 (2053)	7839 (2144)
LC-MICT	8174 (2661)	7957 (1438)	8036 (1289)	8598 (2744)	8463 (2038)	8341 (2427)

Outcome variables are presented as mean (standard deviation). LC-CON: low-carbohydrate diet control group, LC-HIIT: low-carbohydrate diet and high-intensity interval training, LC-MICT: low-carbohydrate diet and moderate-intensity continuous training.
